# Strength and cost analysis of geopolymer concrete using rice husk ash and GGBS as sustainable cement alternatives

**DOI:** 10.1038/s41598-026-43705-3

**Published:** 2026-03-10

**Authors:** Narala Gangadhara Reddy, Veeresh. B. Karikatti, Bheem Pratap, Shabarish V. Patil, Vesna Zalar Serjun

**Affiliations:** 1 School of Building and Civil Engineering, Fiji National University, Samabula, Suva 3722 Fiji; 2 Department of Civil Engineering, K.L.E. Institute of Technology, Hubli, Karnataka 580027 India; 3 Bheem Pratap, Department of Civil Engineering, Graphic Era Deemed to be University, Dehradun, 248002 India; 4 Shabarish V. Patil, Department of Civil Engineering, K.L.E. Institute of Technology, Hubli, Karnataka 580027 India; 5 Vesna Zalar Serjun, Slovenian National Building and Civil Engineering Institute, Ljubljana, 1000 Slovenia

**Keywords:** Geopolymer concrete, Rice husk ash, Ground granulated blast furnace slag, Sulphuric acid, Cost analysis, Structural materials, Environmental impact

## Abstract

The present study aims to develop environmentally friendly concrete by using industrial waste materials, namely ground granulated blast furnace slag (GGBS) and rice husk ash (RHA), to produce green concrete. Geopolymer concrete (GPC) has emerged as an alternative to eliminate the use of cement. The main objective of this study is to design and evaluate geopolymer concrete of M40, M50, and M60 grades. Mixes were prepared by replacing GGBS with RHA at 0%, 10%, 20%, and 30% replacement levels for each grade. The resistance of these mixes against 5% sulphuric acid exposure was also examined. Experiments were conducted to determine compressive strength under different parameters, including NaOH concentration, proportions of RHA and GGBS, and curing duration. Additional tests assessed the effect of acid exposure on strength and weight loss. The cost-effectiveness of GPC production was also compared with that of ordinary Portland cement (OPC). The results revealed that, for all three grades, replacement of GGBS with more than 10% RHA led to a decrease in compressive strength. Furthermore, the production cost of GPC was found to be more economical compared to OPC. Both weight loss and strength loss increased progressively with longer acid exposure. Strength reduction for M40, M50, and M60 grade concretes reached 75.4%, 76.1%, and 79.9%, respectively, when 10% of GGBS was replaced with RHA.

## Introduction

OPC is the most conventional binder for concrete production. A number of efforts are being made to increase the amount of Portland cement used in concrete in order to fight global warming. It is assessed that substituting pozzolans activated by an alkaline solution for Portland cement in solid can decrease CO_2_, emissions by more than 80%^1^. Geopolymer concrete (GPC) is a building material which does not require Portland cement for development of high strength. To develop GPC, the raw material needs to contain alumina, and silicon rich materials as a source for the Si-O-Al bonds. Alkali activated binders are prepared by blending solid alumino-silicate precursors such as fly ash (FA) or GGBS with an alkaline stimulating solution^2^. Higher fineness of fly ash provides more compressive strength; with the increase in fineness, the strength of GPC also increases^3^.

An increase of GGBS percentage level in GPC increases the compressive strength and decreases the workability with the rise in NaOH concentration, the workability of fresh concrete was marginally reduced, and compressive strength rises with an increase in the concentration of NaOH^4^. The maximum flexural and compressive strength was obtained from the 100% GGBS mortars activated by 8% liquid sodium silicate. The compressive strength rises with age of the concrete^5^. Higher silicate modulus (Na₂O/SiO_2_) the strength of the GPC gets increased^6^. The addition of GGBS up to 0% to 30% offers a good result in compressive strength under room temperature curing^7^.

Therefore, industrial by-products have been effectively used in several researches in the past to synthesize geopolymer binders^8^. Because of their exceptional pozzolanic activity and accessibility, fly ash and ground granulated blast furnace slag are the most often utilized precursors^9^. Furthermore, rice husk ash and other agro-waste residues are being investigated as possible precursor materials for geopolymer binders^10^. When rice is ground and its husk is burned as fuel to produce energy, rice husk ash is produced^11^. The amount of rice produced in India in 2022 was 130.29 million metric tons, a 4.76% increase from 2021^6^. A total of 0.28 tons of rice husk are typically created for every ton of rice produced^10^, and when this husk is burned, it loses around 75% of its weight, becoming rice husk ash^12^. Large amounts of rice husk ash have been produced over time, and because of its limited use, it is primarily landfilled. Since land pollution is producing various environmental issues, it is necessary to manage the discarded rice husk ash properly. Concrete strength decreases after 15% addition of RHA in the concrete mix. However maximum strength occurs for 20% FA and 10% RHA replacement in cement^13^. The FA and GGBS greatly diminish the rapid chloride permeability of GPC^14^. Cement replaced by 20% fly ash and 20% GGBS in concrete is more durable in seawater and 1% sulphuric acid solution^15^. It can be concluded that loss in compressive strength and weight are less in GGBS and fly ash based concrete compared to conventional concrete^16,17^. Although extensive research has demonstrated the potential of industrial by-products such as fly ash (FA) and ground granulated blast furnace slag (GGBS) in geopolymer concrete (GPC), several key gaps remain unaddressed. Firstly, most existing studies focus on FA and GGBS as primary precursors, while the use of agricultural waste materials like rice husk ash (RHA) remains relatively underexplored, particularly in ternary or hybrid geopolymer systems. Despite RHA’s high silica content and increasing availability in regions like India, its optimal incorporation levels and effects on long-term durability, workability, and mechanical performance of GPC have not been systematically established.

There is limited research evaluating the combined performance of RHA with FA and GGBS under various curing conditions and aggressive environments such as seawater or acidic exposure. Additionally, while individual impacts of alkaline activator concentrations and silicate modulus on strength development are known, their interactive effects with multi-precursor systems like FA–GGBS–RHA are not well documented. Therefore, a need exists to explore the synergistic use of these by-products in geopolymer formulations and to establish mix design guidelines that balance mechanical strength, workability, environmental impact, and durability performance.

The main aim of the present work is to design and study geopolymer concrete of M40, M50 and M60 grades. The mixes will be designed by replacing 0%, 10%, 20% and 30% of ground granulated blast furnace slag (GGBS) by rice husk ash (RHA) for each grade with various concentrations of NaOH. The prepared samples were tested at various curing ages, and studies were conducted to evaluate the effects of acid exposure on strength and weight loss, with the goal of determining compressive strength and long-term durability. The cost-effectiveness of GPC in comparison to ordinary Portland cement (OPC) concrete production were studied.

## Experimental work

### Materials

GGBS was obtained from Zindal steel industry Bellary Karnataka. It is a powder material which chemical composition is given in Table 1. The specific gravity of GGBS is 2.81. The RHA was obtained from Pranamya biofuel industry, Udupi − 576,124, Karnataka. The specific gravity of RHA is 2.1 and chemical composition is shown in Table 1. RHA, being highly amorphous and silica-rich increase the pozzolanic reactivity and provides reactive sites for geopolymerization, which enhances the formation of gel formation and thus improves the strength of GPC. On the other hand, the CaO-rich GGBS contributes to the formation of C–S–H (calcium silicate hydrate) gel and imparts early strength development. The average particle size of RHA and GGBS is 45 μm and 60 μm respectively. The aggregate is the matrix or structure consisting of relatively inert fine and coarse materials. Crushed angular granite of 20 mm size from a local quarry was used as coarse aggregate (CA). Specific gravity of CA measured as 2.7. The locally available river sand was used as fine aggregate in this study and the specific gravity of fine aggregate measures as 2.8. The acid used in the durability study was concentrated sulphuric acid of 98% purity obtained from local market of Udupi and a density of 1.85 g/cc was utilized to prepare the sulphuric acid solution.

**Table 1:** Chemical composition of GGBS and RHA.

Constituents	GGBS (% wt)	RHA (% wt)
SiO_2_	40.0	87.20
Al_2_O_3_	13.50	0.15
Fe_2_O_3_	1.80	0.16
CaO	39.20	0.55
MgO	3.60	0.35
LOI	1.5	7.64
SO_3_	0.20	0.32
K_2_O	0.20	3.60

### Mix design of GPC

#### Preparation of specimens, casting, and testing

An alkali solution was prepared one day before the specimens. The components of investigated samples were mixed until the binding paste evenly coated all the aggregates and the mixture became uniform. The mixing proportion ratios used for the preparation of samples is shown in Table 2. The homogeneous GPC mixture was cast into cube moulds with dimensions of 150 mm x 150 mm x 150 mm. The casted cubes left to open air (ambient curing) in the laboratory at room temperature 27 ± 2 °C and 65% RH for curing until testing. The GPC grades MG40, MG50, and MG60 were tested at various curing periods 1, 3, 7, and 28 days to evaluate the compressive strength in N/mm². The percentages of RHA used in the mixes were 0%, 10%, 20%, and 30%. The concentration of NaOH solution (in terms of molarity) varied from 6 M to 10 M. For design of MG40, MG50 and Mc60 grades 6 M, 7 M, and 8 M are respectively used. The sodium hydroxide solution was prepared by dissolving flakes of NaOH (pellet form, 98% purity) in distilled water to achieve the desired molarity. The alkali solution was prepared one before the mixing due to its exothermic nature. The mix proportion has been plotted in Table 2. Compressive strength of GPC samples was determined in accordance to IS 516–1959^18^. The effect of acid on compressive strength on GPC sample cubes exposed to 5% solutions of sulphuric acids were compared with unexposed GPC cubes for 30, 45 & 60 days. A pH of 1.5 is maintain for the entire testing period. The pH of the solution is checked for every 5–7 days to maintain a pH of 1.5 during the curing period. Cubes of each grade of GPC were immersed in sulphuric acid solution. The change in weight of geopolymer concrete cubes exposure to 5% sulphuric acid, for test period of 30, 45 and 60 days, were observed and were compared with the weight of unexposed/untreated GPC cubes. The weight losses of GPC cubes exposed to acid were much lower than the unexposed GPC cubes.

**Table 2:** Composition of the samples (M40, M50, and M60 grades of GPC).

Grade of GPC	Mixing proportion ratio							
	Percentage replacement of GGBS with RHA	GGBS (kg/m^3^)	RHA (kg/m^3^)	Fine aggregate (kg/m^3^)	Coarse aggregate (CA) (kg/m^3^)	NaOH (kg/m^3^)	Na_2_SiO_3_ (kg/m^3^)	Extra water (kg/m^3^)
M_G_40	0%	424.6	0	554.4	1293.6	36.9	90.98	45.63
	10%	382.2	42.46					
	20%	339.7	84.92					
	30%	297.2	127.4					
M_G_50	0%	424.6	0	554.4	1293.6	36.39	90.98	45.63
	10%	382.2	42.46					
	20%	339.7	84.92					
	30%	297.2	127.4					
M_G_60	0%	441.6	0	554.4	1293.6	31.54	78.85	26.58
	10%	397.44	44.16					
	20%	353.28	88.32					
	30%	309.12	132.8					

## Results and discussions

### Effect of GGBS and RHA on the compressive strength

**Fig. 1:**
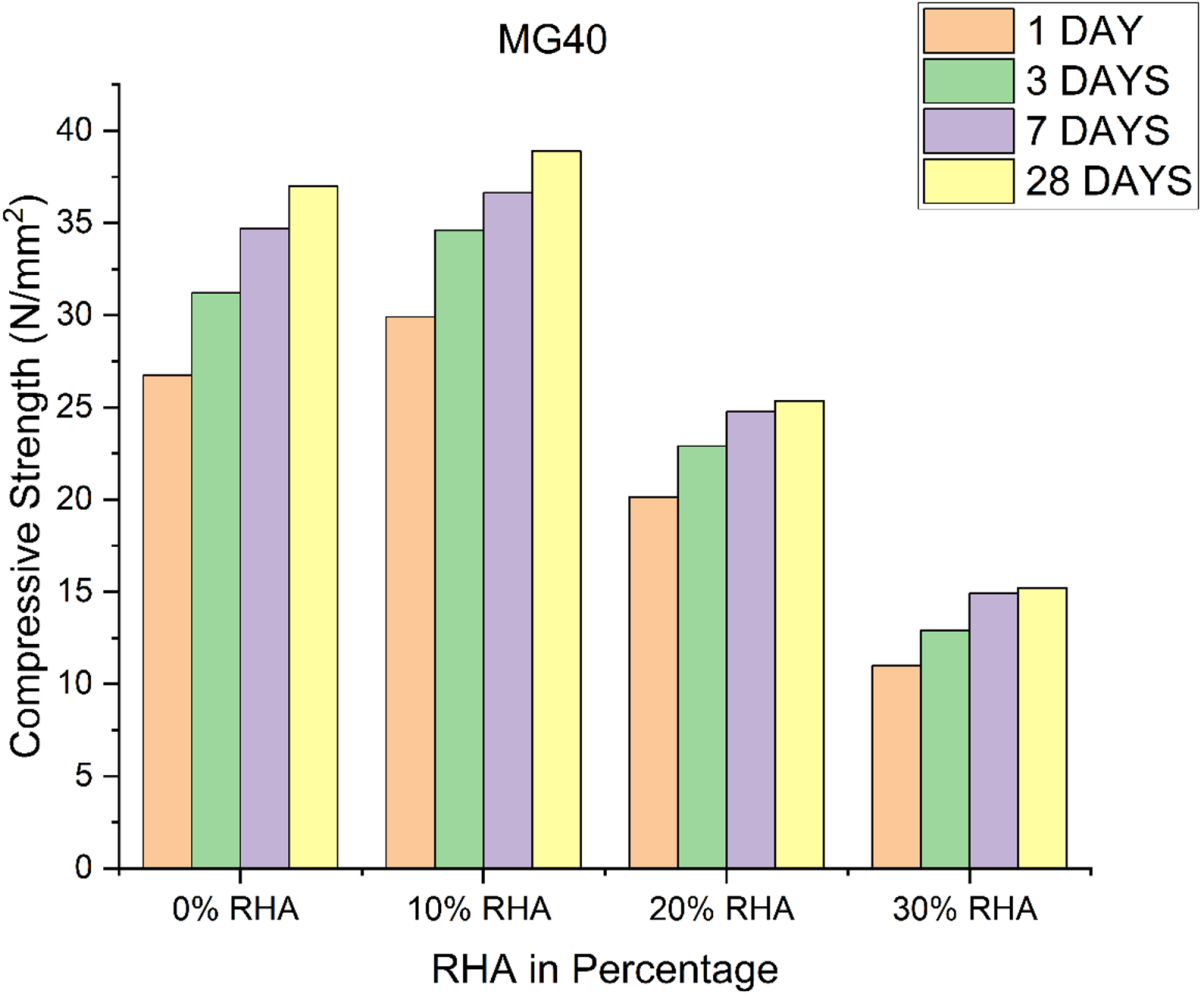
Compressive strength of GPC for different percentage of RHA in M_G_40 grade.

**Fig. 2:**
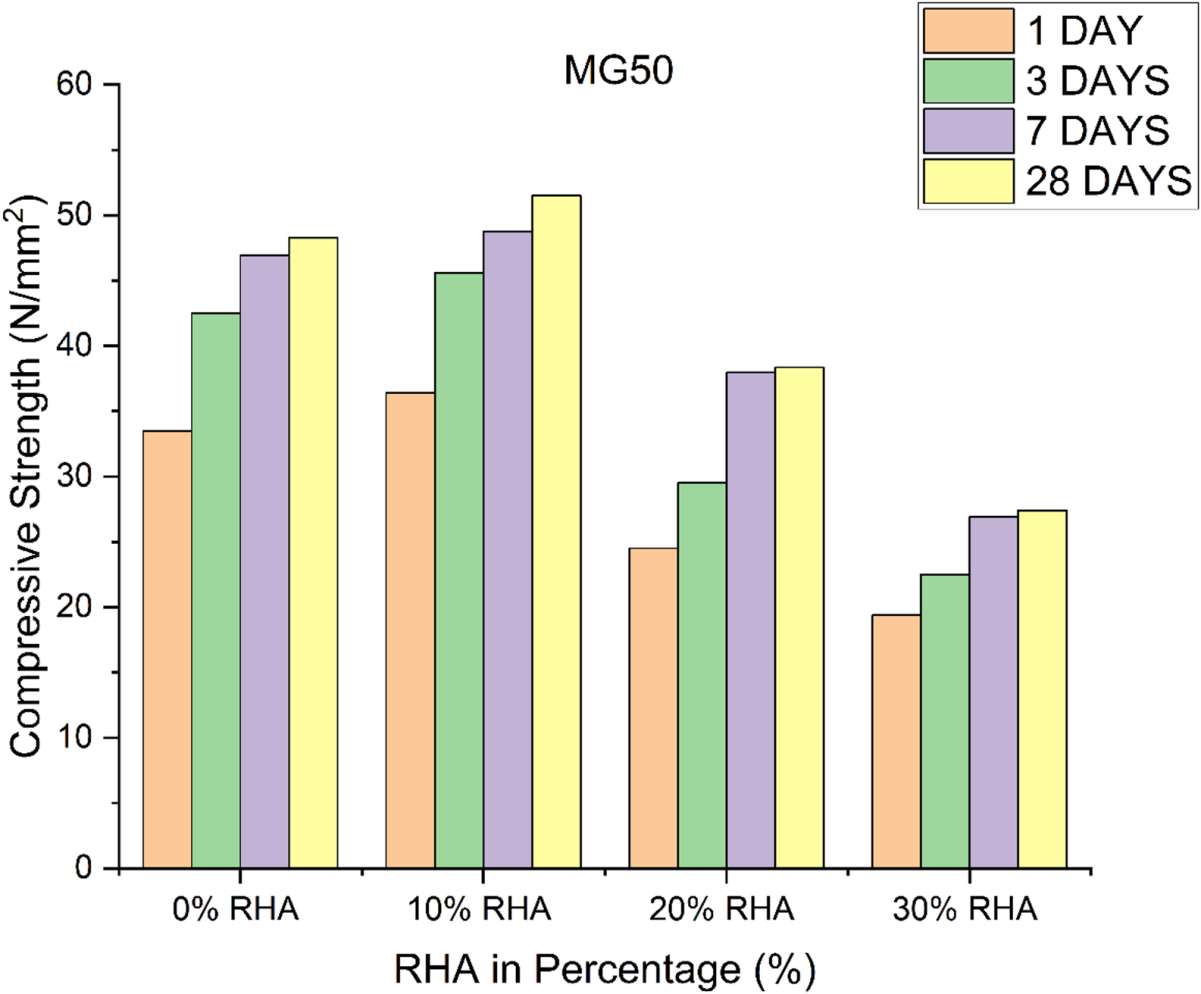
Compressive strength of GPC for different percentage of RHA in M_G_50 grade.

**Fig. 3:**
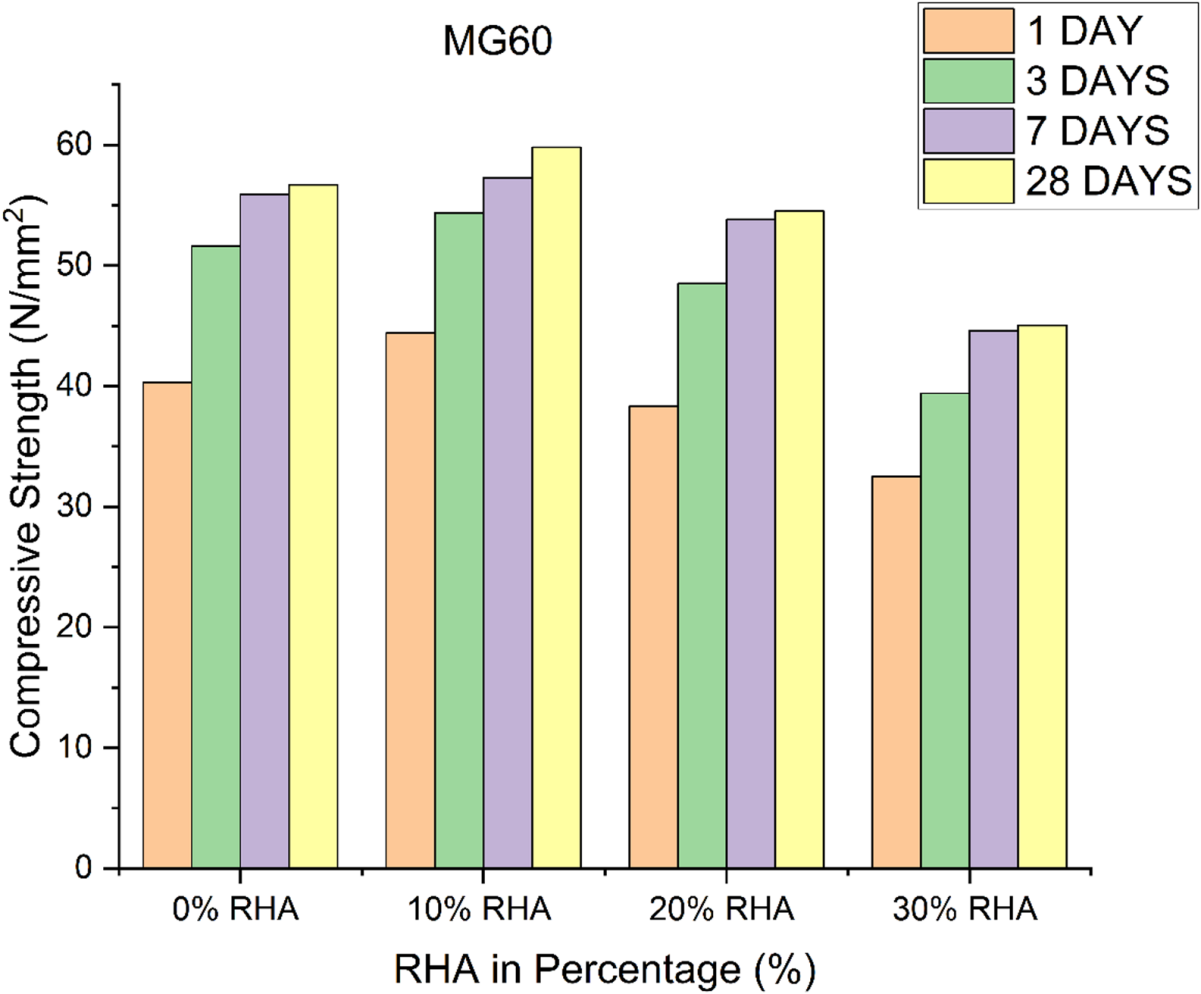
Compressive strength of GPC for different percentage of RHA in M_G_60 grade.

GPC with the highest GGBS contents (90%) possess the highest compressive strength values in all the investigated GPC grades. Figures 1, 2 and 3 presents the compressive strength results of GPC mixes incorporating different percentages of RHA as a replacement for GGBS. In all three grades of GPC (MG40, MG50, and MG60), the compressive strength of the 0% RHA mixes steadily increases over time^19–21^. For instance, in MG40, the compressive strength increases from 26.73 N/mm² at 1 day to 37 N/mm² at 28 days. Similarly, in MG50, the compressive strength rises from 33.5 N/mm² at 1 day to 48.3 N/mm² at 28 days, while MG60 reaches its peak at 56.7 N/mm² by 28 days. This shows that GGBS alone is highly effective in improving the compressive strength of GPC over time, as it provides a continuous reaction and the generation of geopolymeric gel^22^. In comparison to 0% RHA, the compressive strength values rise for all ages when 10% RHA partially substitutes the GGBS. For instance, at day 28, the MG40 mix containing 10% RHA reaches 38.9 N/mm², which is higher than the 37 N/mm² recorded for the 0% RHA mix. This trend holds true for MG50 and MG60 as well, where compressive strengths increase to 51.5 N/mm² and 59.79 N/mm² respectively. The slight enhancement in compressive strength when replacing 10% GGBS with RHA can be attributed to the pozzolanic reaction of RHA, which contributes additional strength in synergy with the GGBS. The finer particle size of RHA improves the microstructure of the geopolymer matrix by filling voids and creating more binding sites^23^.

However, when the RHA content is increased to 20%, the compressive strength of the GPC begins to decline compared to both the 0% and 10% RHA mixes. For example, in the MG40 mix, the compressive strength at day 28 is 25.35 N/mm², which is significantly lower than both the 0% (37 N/mm²) and 10% RHA (38.9 N/mm²) mixes. This trend of strength reduction is consistent in both MG50 and MG60. The decline can be explained by the reduction in the availability of GGBS, which is crucial for the geopolymerization process. Although RHA has pozzolanic properties, its excess replacement at 20% reduces the overall geopolymer matrix’s strength, as the RHA does not provide the same level of calcium content as GGBS for the formation of calcium-silicate-hydrate (C-S-H) gel^24^. At 30% RHA replacement, the compressive strength drops further in all GPC grades. For instance, in MG40, the compressive strength at day 28 is only 15.21 N/mm², a drastic reduction from the 37 N/mm² measured in 0% RHA. In MG50 and MG60, the compressive strength drops to 27.4 N/mm² and 45.03 N/mm², respectively. This significant drop in compressive strength at 30% RHA replacement indicates that excessive RHA content severely compromises the strength development in GPC^25^. At this level, the reduction in GGBS content weakens the geopolymeric binder’s capability, as the RHA itself cannot form enough binding gel for effective strength gain^26^.

A comparison between the mixes shows that with 100% GGBS produces 4% to 6% lower compressive strength compared to the mix with 90% GGBS and 10% RHA. The mixes with GGBS contents reduction from 80% to 70% and increase of RHA 20% to 30%, respectively produced lower strength of about 30% to 60%, respectively compared to the mix with 90% GGBS and 10% RHA. The interaction between GGBS and RHA in GPC is characterized by a balance between the pozzolanic activity of RHA and the geopolymerization potential of GGBS. While small amounts of RHA (10%) improves compressive strength on account to its fineness and pozzolanic reaction, larger replacements (20% and 30%) lead to the contrary effect. The reduction in strength is primarily attributed to GGBS, which provides essential calcium for formation of C-S-H gel, a key component for strength development in GPC^27^. Thus, while RHA can partially replace GGBS, it cannot fully substitute it without compromising the mechanical properties of concrete^28^. GGBS plays a vital role in maintaining the strength of GPC, and the inclusion of RHA as a partial replacement should be carefully optimized to prevent significant strength loss. The results confirmed, that a 10% RHA replacement has a beneficial effect, while higher percentages reduce the compressive strength^21^. To develop strength in GPC, a specific ratio of Si/AI is required. Hence RHA rich in silica content and GGBS rich in aluminium content are added in the concrete. Figures 1, 2 and 3 show that for three different grades of GPC, the compressive strength decreases with increasing RHA content after 10% addition in the GGBS mix. Because Si/Al ratio decreases with increase in RHA content after 10% addition in GGBS mix.

### Effect of time on compressive strength

The 1, 3, 7, and 28 days cube strengths were found out in order to determine the variation in the strength due to the age of curing for three grades of GPC. Compressive strength increases with the age of the geopolymer concrete. For all investigated grade geopolymer concrete (M_G_40, M_G_50 and M_G_60) the compressive strength shows the highest values at 28 days of testing. Figure 3 shows the rise in compressive strength of GPC at 1, 3, 7 and 28 days. Table 3 shows that the rate of increase in compressive strength of GPC is highest at 7 days of curing, and the rate slows down with age. Because the geopolymerization reaction process of the GPC is fast. The maximum compressive strength occurs at 28 days of curing same trend is observed for Mc40, M50 and Me60 grades which is shown in Fig. (1–3).

**Table 3:** Loss of strength caused by acid exposure.

Age (Days)	Substitution of GGBS with RHA (%)	Compressive strength (*N*/mm^2^)								
		Unexposed (*N*/mm^2^)			Exposed (*N*/mm^2^)			Percentage loss (%)		
		M_G_40	M_G_50	M_G_60	M_G_40	M_G_50	M_G_60	M_G_40	M_G_50	M_G_60
**30**	0	33.92	42.1	53.63	33.59	41.8	52.96	1.0	0.8	1.2
	10	36.43	47.8	57.86	36.23	47.51	57.28	0.5	0.6	1.0
	20	25.72	30.1	41.89	24.84	29.16	40.5	3.4	3.1	3.3
	30	17.51	22.2	32.66	16.73	21.15	31.08	4.5	4.9	4.8
**45**	0	34.22	43.5	54.03	33.82	42.72	53.07	1.2	1.7	1.8
	10	37.9	48.1	57.9	37.51	47.59	57.17	1.0	1.1	1.3
	20	25.98	30.5	48.92	24.84	29.13	41.08	4.4	4.5	4.3
	30	18.12	22.6	33.56	17.25	21.52	31.58	4.8	4.9	5.9
**60**	0	35.72	45.4	56.63	34.96	44.33	55.38	2.1	2.4	2.2
	10	38.29	48.9	58.77	37.73	47.87	57.58	1.5	2.1	2.0
	20	26.71	34.5	45.22	25.06	32.29	42.17	6.2	6.4	6.7
	30	19.9	24.1	24.69	18.31	22.1	22.64	8.0	8.2	8.3

### Effect of concentration of sodium hydroxide solution on compressive strength

To the effect of different concentrations of sodium hydroxide solution on the compressive strength of geopolymer concrete M40, M50 and M60 grades is shown in Table 4.

**Table 4:** Percentage weight loss of GPC cubes on acid exposure.

% Of RHA replacement in GGBS	Initial Weight of specimen (kg)			Weight of specimen after 60 days (kg)			Weight loss of specimen after 60 days (%)		
	M_G_40	M_G_50	M_G_60	M_G_40	M_G_50	M_G_60	M_G_40	M_G_50	M_G_60
0	8.2	8.32	8.4	8.01	8.3	8.38	2.32	2.40	2.38
10	8.15	8.32	8.27	8.01	8.17	8.11	1.72	1.80	1.93
20	7.75	7.64	7.7	7.5	7.39	7.45	3.23	3.27	3.25
30	7.3	7.47	7.25	6.89	7.09	6.87	5.62	5.09	5.24

**Table 5:** Cost of production of GPC.

RHA in GGBS	Materials	Rate per kg in (Rs)	Quantity (kg/m^3^)	Cost	Total quantity	Cost (Rs)	Total quantity for M_G_60	Cost (Rs)
			For M_G_40	(Rs)	For M_G_50			
**0% RHA**	Coarse Aggregate	0.85	1293.6	1099.5	1293.6	1099.5	1293.6	1099.5
	Fine Aggregate	0.95	554.3	526.58	554.3	526.58	554.3	526.58
	GGBS	2.00	424.61	849.22	424.61	849.22	441.6	883.2
	RHA	1.00	0	0	0	0	0	0
	NaOH	10.00	90.98	909.8	90.98	909.8	78.85	788.5
	Na_2_SiO_3_	25.00	36.39	909.75	36.39	909.75	31.35	783.75
	**Total cost in Rs.**			**4294.9**		**4294.1**		**4081.5**
**10% RHA**	Coarse Aggregate	0.85	1293.6	1099.5	1293.6	1099.6	1293.6	1099.6
	Fine Aggregate	0.95	554.3	526.5	554.3	526.55	554.3	526.55
	GGBS	2.00	382.15	764.3	382.15	764.3	397.44	794.88
	RHA	1.00	42.46	42.46	42.46	42.46	44.16	44.16
	NaOH	10.00	90.98	909.8	90.98	909.8	78.85	788.5
	Na_2_SiO_3_	25.00	36.39	909.7	36.39	909.75	31.6	790.0
	**Total cost in Rs.**			**4252.4**		**4252.5**		**4043.6**
**20% RHA**	Coarse Aggregate	0.85	1293.6	1099.5	1293.6	1099.6	1293.6	1099.5
	Fine Aggregate	0.95	554.3	526.58	554.3	526.58	554.3	526.58
	GGBS	2.00	339.69	679.38	339.69	679.38	353.28	706.56
	RHA	1.00	84.92	84.92	84.92	84.92	88.32	88.32
	NaOH	10.00	90.98	909.8	90.98	909.8	78.85	788.50
	Na_2_SiO_3_	25.00	36.39	909.75	36.39	909.75	31.60	790.0
	**Total cost in Rs.**			**4209.9**		**4209.9**		**3999.5**
**30% RHA**	Coarse Aggregate	0.85	1293.6	1099.5	1293.6	1099.6	1293.6	1099.5
	Fine Aggregate	0.95	554.3	526.58	554.3	526.58	554.3	526.58
	GGBS	2.00	297.23	594.46	297.23	594.46	303.12	618.24
	RHA	1.00	127.3	127.3	127.3	127.3	132.48	132.48
	NaOH	10.00	90.98	909.8	90.98	909.8	78.85	788.5
	Na_2_SiO_3_	25.00	36.39	909.75	36.39	909.75	31.6	790
	**Total cost in Rs.**			**4167.4**		**4167.5**		**3955.3**

**Fig. 4:**
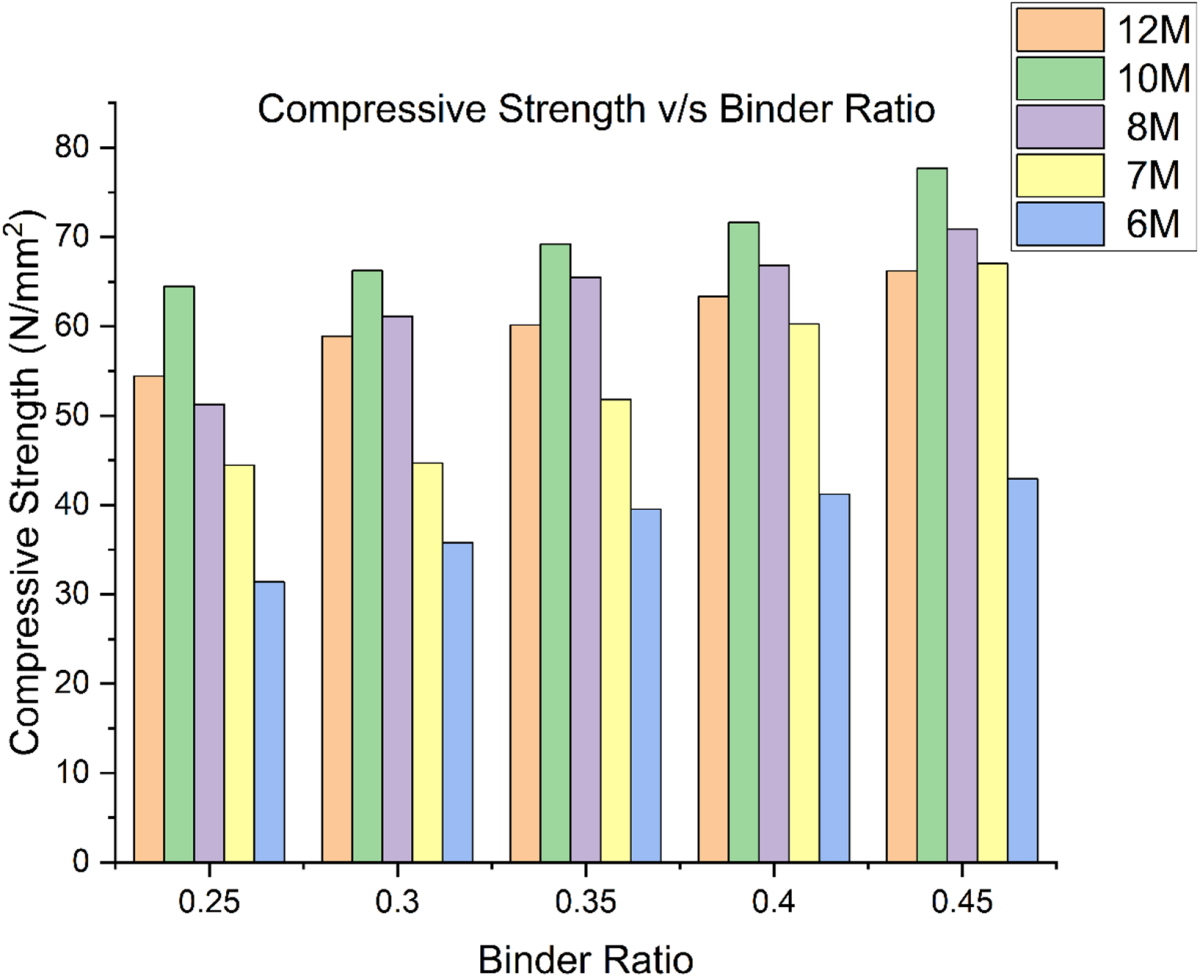
Compressive strength of GPC for different molarities.

Hence in geopolymer synthesis, both the microstructure and compressive strength of geopolymers are strongly impacted by the sodium hydroxide content. For 10 M molar concentration, as seen in Fig. 4, a higher compressive strength is attained due to the increased dissolving of the original solid materials and the increased geopolymerization process caused by the use of high concentrations of sodium hydroxide^21^. Figure 4 presents the relationship between binder ratio and compressive strength of concrete for different molarities of alkaline solutions, typically used in geopolymer concrete. The compressive strength reaches 54.42 N/mm² for a binder ratio of 0.25 and increases with higher binder ratio, reaching 66.2 N/mm² at a binder ratio of 0.45. This indicates a strong positive correlation between binder ratio and strength at this high molarity level, suggesting that more binder results in a better reaction with the alkaline activator, thus enhancing the concrete’s strength^21^. At 10 M, the compressive strength reaches higher values than that of 12 M (64.44 N/mm² at a binder ratio of 0.25). It increases gradually to 77.69 N/mm² at a binder ratio of 0.45, indicating that even at a slightly lower molarity; the concrete still demonstrates a strong capacity for strength improvement with increased binder ratio. In this case, the compressive strength sees a significant increase as the binder ratio rises. At a binder ratio of 0.25, the strength is 51.24 N/mm², and by the time the ratio reaches 0.45, it grows to 70.9 N/mm². At 10 M molarity, the strength values at the lowest binder ratios are lower compared to higher molarities (starting at 44.45 N/mm² for a binder ratio of 0.25). However, the compressive strength increases substantially with the binder ratio, reaching 67.02 N/mm² at the 0.45 ratio, indicating that even for lower molarities, increasing binder content has a significant positive impact on the material’s strength^21^. For the lowest molarity (6 M), the compressive strength values are consistently lower than the others, starting at 31.36 N/mm² for the 0.25 binder ratio and reaching 42.91 N/mm² at the 0.45 ratio. While the trend of increasing strength with higher binder ratio holds true, the overall strength is much lower compared to higher molarities. The result suggest that higher binder ratios and higher molarities of alkaline solutions generally lead to increased compressive strength in geopolymer concrete^16^. The 10 M solutions exhibit the highest strength values from all binder ratios, whereas lower molarity solutions (like 6 M and 7 M) show less strength development even with higher binder ratios.

### Effect of acid on compressive strength

The percentage compressive strength loss of three different grades of GPC for different percentage level of RHA usage in GGBS is tabulated in Table 3.

Table 3 indicates loss in compressive strength of cubes containing 0%, 10%, 20% and 30% RHA respectively exposed to acid for 30 days shows 1.0%, 0.5%, and 3.4% and 4.5% respective loss in compressive strength in comparison to unexposed GPC cubes. Similarly GPC cubes exposed to acid for 45 days and 60 days shows 1.2%, 1.0%, 4.4%, 4.8% and 2.1% 1.5%, 6.2% and 8.3% respective strength losses in comparison to unexposed GPC cubes. Table 3 shows that for all three grades of GPC with a 10% GGBS replacement by RHA, the minimum strength loss observed was approximately 2% after 60 days of acid exposure. With the increasing of the exposure time (from 30 to 60 days), the unexposed compressive strength generally increases for all concrete grades and RHA replacement percentages. Exposure to sulfuric acid results in a loss of compressive strength in all cases, though the extent of this loss varies with the RHA replacement percentage and the age of the concrete^29^. For 0% RHA replacement, the compressive strength in unexposed cubes steadily increases as the curing age increases. MG60 mix, the compressive strength increases from 53.63 N/mm² at 30 days to 56.63 N/mm² at 60 days. The compressive strength of exposed cubes for the same mix also shows a slight decrease after sulfuric acid exposure, with a percentage loss that rises as the curing age increases (e.g., from 1.2% at 30 days to 2.2% at 60 days)^16^.

10% RHA replacement shows similar trends, but the compressive strength of unexposed cubes is higher than 0% RHA, particularly at 30 days and 45 days, indicating potential benefits of using RHA for initial strength gain. However, exposed cubes exhibit a small percentage loss, remaining relatively low between 0.5% and 2.1%. With 20% RHA replacement, the compressive strength of unexposed cubes decreases notably compared to 10% RHA, particularly at 30 days (e.g., 41.89 N/mm² for MG60). Exposed cubes also showed greater strength loss after sulfuric acid exposure, with the percentage loss rising from 3.3% at 30 days to 6.7% at 60 days. 30% RHA replacement leads to further reduction in the unexposed compressive strength, and exposed cubes show the highest percentage loss^30^. At 60 days, the percentage loss is 8.3% for MG60, suggesting that higher RHA replacement may reduce resistance to sulfuric acid attack.

At 30 days, the compressive strength of unexposed cubes is lower, but the percentage loss in strength after sulfuric acid exposure is also relatively small (e.g., 1.0%–1.2% for 0% RHA). At 45 and 60 days, while the unexposed strength increases, the percentage loss after exposure to acid becomes more significant, especially for higher RHA replacement levels. This suggests that longer curing times provide greater initial strength but may also lead to greater vulnerability to acidic environments over time, particularly with higher RHA content^31^. The results illustrate the complex interactions between RHA replacement levels, curing age, and sulfuric acid exposure on the compressive strength of GGBS concrete. Lower percentages of RHA (0%–10%) result in relatively small strength losses after acid exposure, while higher RHA levels (20%–30%) lead to significant compressive strength reduction.

### Effect of acid on weight loss

Table 3 appears to focus on the effect of replacing GGBS with RHA in different proportions, specifically 0%, 10%, 20%, and 30%, on the weight loss of concrete specimens over a 60-day period. The columns represent three different concrete mixtures—labelled MG40, MG50, and MG60—where the numbers indicate the concrete grade based on strength. Each row within the table represents the data for a specific percentage of RHA replacement in GGBS, while the columns provide the initial weight of the specimen, the weight after 60 days, and the corresponding weight loss percentage.

At 0% RHA replacement (i.e., 100% GGBS), the initial weights of the concrete specimens are comparable, ranging from 8.2 kg (MG40) to 8.4 kg (MG60). After 60 days, the weight loss is between 2.32% and 2.40%, with minor variations within the different mixtures. The relatively low weight loss suggests that 100% GGBS concrete is stable under the conditions of the experiment. When 10% of the GGBS is replaced with RHA, the initial weights of the specimens remain fairly consistent with the 0% replacement condition, suggesting that the inclusion of RHA has not significantly altered the bulk mass^32^. However, after 60 days, the weight loss percentages are lower than those of the 0% replacement specimens, ranging from 1.72% (MG40) to 1.93% (MG60). This reduction in weight loss indicates that a small RHA replacement improves the durability of the concrete, likely due to RHA’s ability to enhance the concrete’s resistance to degradation. For a 20% RHA replacement, the initial weights of the specimens are slightly lower than 10% RHA ranging from 7.64 kg (MG50) to 7.75 kg (MG40). The weight loss percentages, however, are higher than those observed for the 10% replacement samples, with values between 3.23% and 3.27%. This increase in weight loss suggests that replacing 20% of GGBS with RHA reduces the concrete’s stability, possibly due to the lower bonding properties of RHA at higher percentages^33^. At 30% RHA replacement, the initial weights of the specimens decrease further, with MG40 weighing 7.3 kg and MG60 weighing 7.25 kg. The weight loss after 60 days is significantly higher compared to all other RHA replacement levels, with values ranging from 5.09% (MG50) to 5.62% (MG40). These results suggest that replacing 30% of GGBS with RHA may negatively impact the durability of the concrete, possibly due to the reduced pozzolanic activity or increased porosity at higher RHA content^23,34–37^.

**Fig. 5:**
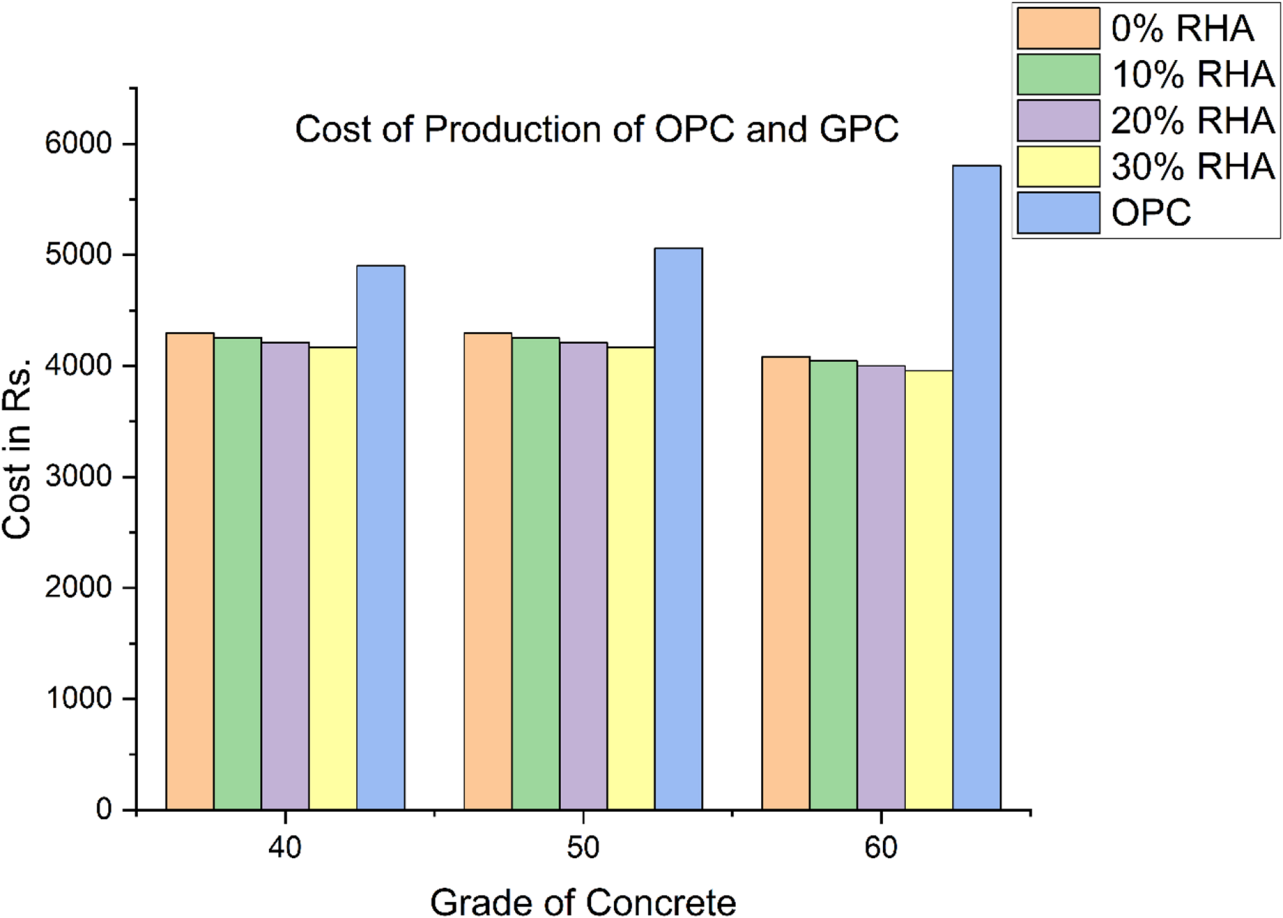
Cost of production of three different grades of OPC and GPC.

The results have shown that replacing GGBS with RHA in GPC has a variable effect on weight loss over time, with 10% RHA showing the most promising results in terms of durability. Higher RHA replacement levels (20% and 30%) lead to increased weight loss, indicating reduced durability, due to the limitations in RHA’s contribution to the concrete’s strength and stability^38^. The geopolymer concrete showed minor surface and edge damage on the specimens, but the overall extent of the damage was considerably reduced. The weights of the cubes made up of different percentages of RHA in GGBS were found to be less. Table 4 clearly shows that the weight loss of M-40, M50 and M.60 grade increases with increase in percentage of RHA in GGBS and period of exposure of cubes in acid. In the present study cubes made up of 10% RHA and 90% GGBS exposed to acid for 60 days shows minimum weight loss of 1.72%, 1.8% and 1.93% were observed from Table 6 for M40, M50 and M60 grades respectively. Hence it can be concluded from Fig. 5 that the minimum weight loss of GPC cubes for all three grades occurs at 10% RHA in GGBS. The results confirmed that the low calcium hydration products were more durable in the acid environment^21,39^.

**Table 6:** Cost of production of OPC concrete for three different grades.

Grade of OPC	Materials	Cost per kg	Quantity in kg	Total cost in Rs
M40	Cement	8.2	400	3280
	Coarse Aggregate	0.85	1168	992.8
	Fine Aggregate	0.95	660.2	627.19
	**Total cost in Rs.**			**4899.99**
M 50	Cement	8.2	412	3378.4
	Coarse Aggregate	0.85	1284	1091.4
	Fine Aggregate	0.95	621	589.95
	**Total cost in Rs.**			**5059.75**
M60	Cement	8.2	510	4182
	Coarse Aggregate	0.85	1043.8	887.23
	Fine Aggregate	0.95	773	734.35
	**Total cost in Rs.**			**5803.58**

### Cost analysis

The estimated material cost was obtained from Local suppliers. A description of the item, size, quantities, and actual prices of the materials are listed below in Tables (5–6).

From strength point of view 10% of GGBS replaced by RHA in M40, M50 and M 60 grades of GPC are considered for cost effectiveness. Hence the overall savings in cost of production for M-40, M50 and M 60 is 13.1%, 15.9%, and 30.3% respectively over OPC concrete of M40, M50 and M60 grades. Therefore, it can be concluded that cost savings are achievable in the production of higher-grade GPC Table 8 clearly indicates that the cost of cement is higher compared to the cost of GGBS and RHA used for production of all three grades. From a strength perspective, replacing 10% of GGBS with RHA allows for savings of 75.4%, 76.1%, and 79.9% in the production of M40, M50, and M60 grade concrete, respectively.

**Fig. 6:**
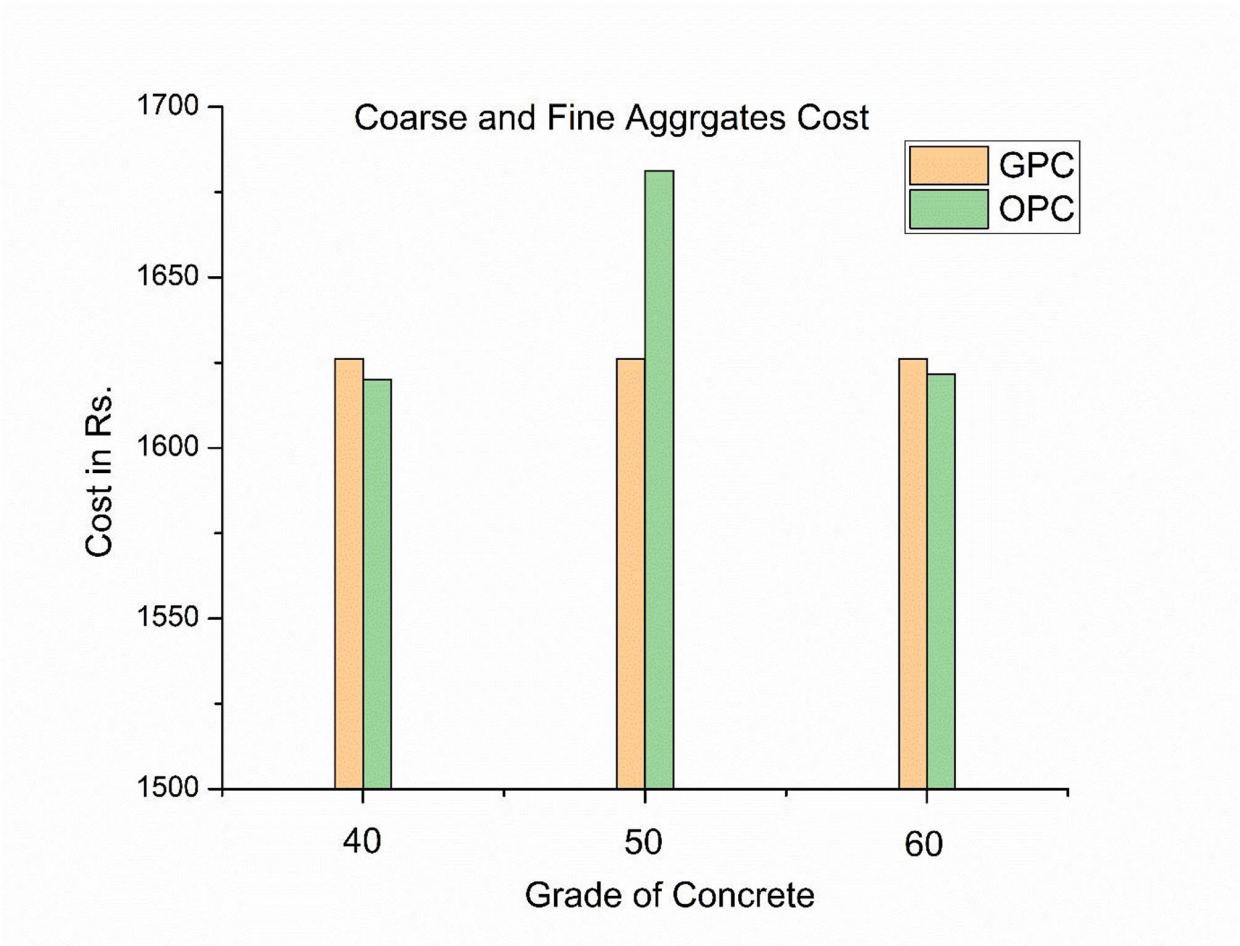
Cost of aggregate used in production of three different grades of OPC and GPC.

The cost of the coarse and fine aggregates required to produce geopolymer concrete for the three grade levels evaluated in Fig. 6 is Rs. 1653.86. Additionally, the manufacturing costs for M40, M50, and M60 grade OPC concrete were Rs. 1619.99, 1681.35, and 1621.58, respectively. Since GPC concrete contains more coarse aggregate than OPC concrete, its aggregate production cost is slightly higher, as shown in Fig. 5. Because as per mix design assuming 70% to 80% of density of concrete which is constant in GPC, but in normal OPC concrete mix design it depends on IS code 10,262 − 2009. Figure 6 shows that cost of an alkaline solution required for production of geopolymer concrete is Rs 1819.55 same for both M40 and M50 grades since the binder ratio is 0.3 for both the grades and for M 60 grade cost is Rs 1572.25. The cost of alkaline solution decreases with increase in grade of geopolymer concrete.

## Conclusions

The experimental program was designed to evaluate the fresh, mechanical, and durability properties of geopolymer concrete incorporating varying proportions of Rice Husk Ash (RHA) and Ground Granulated Blast Furnace Slag (GGBS) across different concrete grades. The study focused on understanding the influence of RHA content, GGBS ratio, and alkaline activator concentration on workability, strength, and acid resistance. Based on the experimental investigations and analysis of results, the following conclusions are drawn:

- The workability of the geopolymer concrete decreases with an increase in the grade of the concrete. For M40, M50, and M 60 the replacement of GGBS by RHA up to 10% gave sufficient workability. With an increase in the percentage of RHA content after 10% in GGBS workability of geopolymer concrete decreases.
- Compressive strength increases with the increase in the percentage of GGBS i.e. from 70% to 100% and RHA replacement up to 10% in geopolymer. Compressive strength decreases with increase in the percentage of RHA above 10%.
- Higher compressive strength is achieved at 28 days of testing of cubes of different percentage of RHA replacement in GGBS mix for all three grades of GPC. Higher concentration (8 M) of sodium hydroxide solution results in higher grade (M60) of geopolymer concrete.
- Increase in exposure period of GPC cubes to sulphuric acid decreases the compressive strength and weight of the cubes. Compressive strength of GPC cubes prepared with 10% RHA and 90% GGBS on exposure to 5% sulphuric acid for 60 days showed minimum strength loss of about 2% compared to the ambient cured cubes.
- Weight loss is nearly 1.9% in case of GPC cubes made up of 10%RHA and 90%GGBS for all three grades exposed to acid for 60days in comparison to unexposed GPC cubes. Increase in percentage of RHA up to 30% in GGBS reduces the weight of the GPC cubes by 10%.
- The production cost of GPC is lower and more economical compared to that of ordinary Portland cement (OPC) concrete. The use of RHA and GGBS in the production of GPC concrete reduces the cost of cement. Aggregate usage is more in GPC production compared to OPC concrete production. Hence overall cost of geopolymer concrete production is less compared to OPC concrete.

Further work will include more detailed microstructural and phase compositional characterization (e.g., SEM, XRD, FTIR) to provide deeper insights into the formation and distribution of newly formed phases, such as geopolymer gels, and more straightforward insight into the correlation between microstructural features and mechanical features (strength). Investigation into the chemical mechanisms associated with NaOH molarity on compressive strength will also be undertaken. Benchmarking the durability results under acid exposure against existing literature is foreseen to strengthen the contextual understanding of performance. Future work will also extend the cost analysis by incorporating broader environmental aspects, such as reduced carbon footprint and waste management benefits. In addition, a quantitative life-cycle assessment (LCA) and carbon emission analysis are planned to more comprehensively demonstrate the sustainability potential of using RHA and GGBS.

## Data Availability

The datasets used and/or analysed during the current study available from the corresponding author (Narala Gangadhara Reddy) on reasonable request.
